# The Expression of IL-17, in Chronic Spontaneous Urticaria Is Linked to Semaphorin5A

**DOI:** 10.3390/biom11030373

**Published:** 2021-03-02

**Authors:** Matanis Lobna, Eiza Nasren, Sabag Adi, Bejar Jacob, Gimenez-Arnau Ana Maria, Maurer Marcus, Vadasz Zahava

**Affiliations:** 1 The Proteomic and Clinical Flow Cytometry Unit, Bnai-Zion Medical Center, The Rappaport Faculty of Medicine, Technion, Haifa 31048, Israel; lobna.w.m@gmail.com (M.L.); dr.niss@gmail.com (E.N.); adi.sabag@b-zion.org.il (S.A.); 2 Bnai-Zion Medical Center, The Division of Pathology, Haifa 31048, Israel; jacob.bejar@b-zion.org.il; 3 Department of Dermatology, Hospital del Mar, IMIM, Universitat Autonoma, 08016 Barcelona, Spain; anamariagimenezarnau@gmail.com; 4 Dermatological Allergology, Allergie-Centrum-Charite, Department of Allergy and Dermatology, Charite-Universitätsmedizin, 12529 Berlin, Germany; marcus.maurer@charite.de

**Keywords:** chronic spontaneous urticaria, T cells, mast cells, semaphorin5A, IL-17

## Abstract

**Background:** Patients with chronic spontaneous urticaria (CSU), an autoimmune disorder, show increased skin expression of IL-17A and can benefit from treatment with the anti-IL-17A biologic secukinumab. The mechanisms that drive IL-17A expression in CSU are currently unknown, but may involve Semaphorin5A (Sema5A). **Objective:** To explore the expression, role, and effects of Sema5A in CSU and its link to IL-17A. **Material and Methods:** We investigated patients with CSU and healthy controls for skin expression of expressing peripheral T cells. **Results:** Sema5A was highly expressed in the skin of CSU patients as compared to healthy control skin. Both CD4+ T cells and mast cells in CSU skin expressed Sema5A, and many of them expressed both Sema5A and IL-17A. Patients with CSU had significantly higher rates of IL-17A-expressing CD4+ T cells as compared to healthy controls. Incubation with Sema5A increased the rates of IL-17A-expressing CD4+ T cells in healthy controls to CSU levels. **Conclusion:** Sema5A may drive the expression and effects of IL-17A in CSU. Further studies in larger cohorts are needed to confirm the role of Sema5A in the pathogenesis of CSU and to explore its potential as a therapeutic target.

## 1. Introduction

Chronic spontaneous urticaria (CSU), in most patients, is an autoimmune disorder that is driven by the activation of skin mast cells by autoantibodies. In some patients, IgE autoantibodies against thyroid peroxidase and other auto-allergens are detected and held to induce mast cell activation and degranulation when they engage them. This endotype is defined as type I autoimmune CSU. In others, CSU is characterized by activating IgG and IgM autoantibodies directed against the high affinity IgE receptor, FcεRIα, on skin mast cells and basophils. This endotype is defined as type IIb autoimmune CSU [1,2,3].

The notion that CSU is an autoimmune disease is supported by the key role that T cells play in its pathogenesis. Activated CD4+ T cells and auto-reactive T cells directed against FcεRIα are found in the peripheral blood as well as in the skin of CSU patients. This may explain why patients with CSU respond to immunosuppressive therapies, for example glucocorticoids or cyclosporine [4,5,6]. In a very recent study, we demonstrated that the expression of interleukin-17A (IL-17A), a cytokine linked to many autoimmune diseases including rheumatoid arthritis, systemic lupus erythematosus, psoriasis, and multiple sclerosis, is increased in the skin of patients with severe CSU. This is in line with previous studies that reported increased levels of IL-17A in the peripheral blood of patients with severe CSU. We also showed that secukinumab, an anti-IL-17A antibody licensed for the treatment of psoriasis, can be effective in the treatment of patients with severe CSU [7]. As of now, the drivers of IL-17A expression in CSU remain unknown.

Semaphorin5A (Sema5A) is member of the semaphorin family and considered to be one of the “immune semaphorins” because of its role in driving both innate and adaptive immune responses [8]. Sema5A promotes T cell and NK cell proliferation and increases the secretion of T helper type I (Th1/Th17) pro-inflammatory cytokines including IL-17. High levels of soluble Sema5A are found in the serum of patients with rheumatoid arthritis, where IL-17A triggers changes in the synovium that lead to synovitis and maintain local inflammation [9]. Elevated blood levels of Sema5A levels are also found in patients with systemic lupus erythematosus where IL-17 importantly contributes to the promotion and perpetuation of inflammation and the damage to affected tissues. Blood levels of Sema5A are also increased in patients with chronic immune thrombocytopenia and are positively correlated with disease activity, higher levels of IFN-ϫ, and with Th1 polarization [10,11]. Based on these findings, Sema5A may be a driver of IL-17A expression in patients with CSU.

Here, we assessed the expression of Sema5A in the skin of patients with CSU and investigated its link to skin mast cells, CD4+ T cells, and IL-17A. Also, we analyzed the effects of Sema5A on IL-17A expression in peripheral CD4+ T cells in patients with CSU and healthy controls.

## 2. Material and Methods

### 2.1. Study Subjects and Ethical Considerations

We analyzed samples of 30 patients with anti-histamine-refractory CSU and 10 healthy control subjects from a study recently reported [7]. The study was approved by our local ethical committee and all studied individuals signed an informed consent.

### 2.2. Immunohistochemistry

Skin biopsies from lesional CSU patients were assessed for the expression levels of Sema5A and IL-17A as well as CD4+ T cells and mast cells, each alone and double-stained for Sema5A. The same biopsies were also assessed for co-expression of Sema5A and IL-17A. Briefly, formalin-fixed paraffin embedded skin tissue sections were serially cut into 5-µm slices by a microtome. The slides were deparaffinized by xylene and then rehydrated by absolute ethanol, 96% ethanol, 70% ethanol, and two short distilled water washes. Antigen retrieval was done by cooker pressure in an EDTA solution, ph 8.0. In order to block non-specific endogenous biotin, we used a commercial biotin blocker, according to the manufacturer’s instructions. Slides were incubated with primary monoclonal anti human sema5A antibody (1:50 monoclonal mouse IgG1 clone #914419, R&D systems-for the immunohistochemistry and for double staining-goat anti-human Sema5A-Abcam, 1:100), rabbit polyclonal anti-human IL-17A (1:100, Proteintech group LTD-for the immunohistochemistry and for double staining-rabbit anti-human Sema5A-BioRad, 1:500), rabbit polyclonal anti-human cKit (Abcam1:100), and rabbit polyclonal anti-human CD4 (Abcam1:300). Secondary antibody staining was performed by using the “Histostain+” kit (Invitrogen, Camarillo, CA, USA) according to the manufacturer’s instructions. Isotype control antibodies and slides that underwent the same procedure but without primary or secondary antibodies were used as negative controls. The immune-histochemical staining was evaluated by two independent pathologists and the localization and staining intensity were assessed as previously described [8]. The extent of staining was given a score from 0 to 3 (0 = absent, 3 = strong). Numbers of CD4+ T cells and cKit+ (CD117+) mast cells in skin biopsies each alone and double-stained for CD4+/IL-17A, CD4+/Sema5A, CD117+/IL-17A, CD117+/Sema5A, and IL-17A/Sema5A were assessed by quantitative histomorphometry (IMARIS microscope software). Using this highly accepted method of quantification, whole microscopic slides were scanned, and numbers of CD4+ and CD117+ cells expressing IL-17A and/or Sema5A per microscopic high-power field (HPF) were calculated.

### 2.3. Peripheral Activated CD4+ T Cell Assays

Peripheral blood was obtained from 10 healthy individuals and 12 patients with severe CSU treated at the Division of Allergy & Clinical Immunology of Bnai-Zion Medical Center, Haifa, Israel. All patients had severe CSU refractory to high off-label doses of H1-anti-histamines and short courses of steroids. Peripheral blood mononuclear cells (PBMCs) were isolated on lymphoprep, and CD4+ T cells were then purified by positive selection on magnetic columns using anti-human CD4 microbeads (Miltenyi Biotec, Bergisch Gladbach, Germany) according to the manufacturer’s instructions, achieving >97% purity. Purified CD4+ T cells from both healthy individuals and CSU patients were then activated in a 12-well cell culture plate with anti-human CD3 monoclonal antibodies (UCTHT1) and anti-human CD28 antibodies (eBioscience, San Diego, CA, USA). Recombinant human Sema5A (R&D) at 5 µg/mL was either added or not (control cells) to these activated T cells. Cells were incubated at 37 °C for 48 h. At the day of the experiment, cells were seeded and resuspended in 200 µL PBS, and anti CD4-FITC (A07750, Beckman Coulter) was added for 30 min at room temperature. Afterwards, cells were fixed with 100 µL Fix and Perm medium A (GAS001, Life Technology, Carlsbad, CA, USA) for 10 min. This was followed by washing and permeabilization with 100 µl Fix and Perm medium B (GAS021, Life Technology), and anti hIL-17 APC (IC3171A, R&D) was added. Stained cells were incubated at room temperature for an extra 30 min, and then they were washed and resuspended in 350 µL PBS. The staining was evaluated in NAVIOUS EX Flow Cytometer, and the results were analyzed using Kaluza, Flow Cytometry Analysis Software 2.1.

### 2.4. Statistical Analysis

Data are expressed as mean ± stand error of the mean. Comparisons of the differences between two groups were performed using the unpaired two tailed Student t-test, whereas for more than two groups it was done by using ANOVA test. For populations with non-normal distribution, we used the non-parametric Mann–Whitney test. Statistical significance was considered with a *p*-value of 0.05 or less. For all the statistical tests we used GraphPad Prism 9.0.1 software.

## 3. Results

### 3.1. Many CD4+ T Cells and Mast Cells in the Skin of CSU Patients Express Sema5A and IL-17A

**Figure 1:**
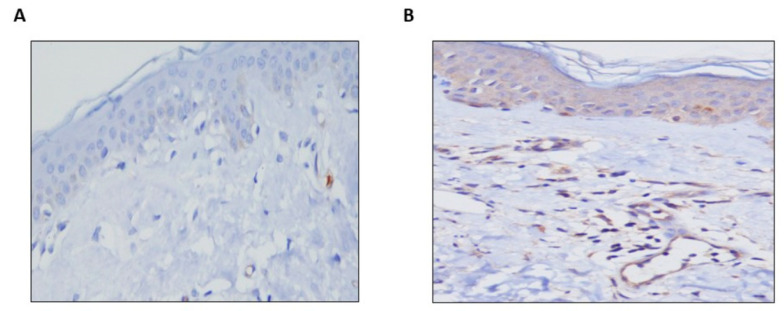
Semaphorin5A expression is increased in the lesional skin of patients with CSU. Almost no Sema5A expression is seen in the skin of healthy control subjects (**A**) as compared to strong expression in the skin of CSU patients (**B**). The pictures are 20× magnification.

Expression levels of Sema5A in the lesional skin of CSU patients were significantly higher (mean score 2–3) as compared to the skin of healthy individuals, where Sema5A expression was almost absent (mean score 0–1; *p* < 0.0001; Figure 1).

**Figure 2:**
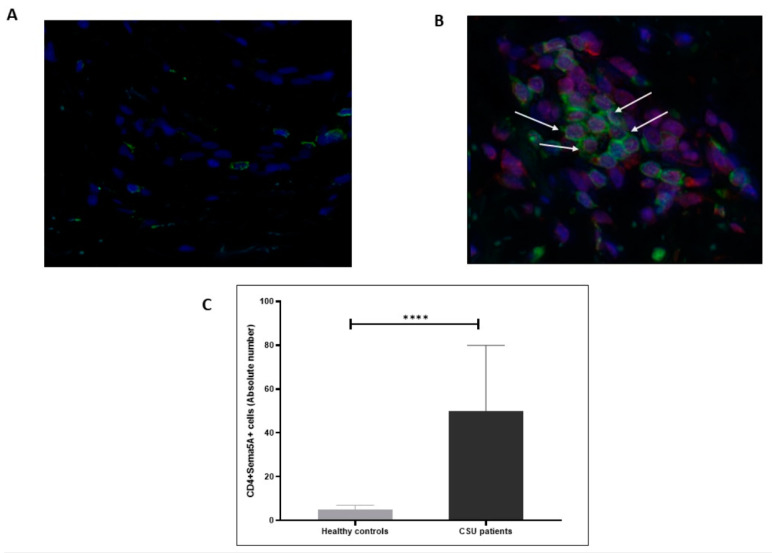
CD4+ T cells in the lesional skin of patients with CSU express Semaphorin5A. Sema5A is expressed by CD4+ T cells (**B**, white arrows) in the skin of CSU patients compared to healthy controls (**A**). (**C**) shows the results of quantitative analysis of the double-staining of Sema5A/CD4+ T as evaluated by IMARIS software (as detailed in the “Methods” section). The pictures are 40× magnification. **** statistically significance.

**Figure 3:**
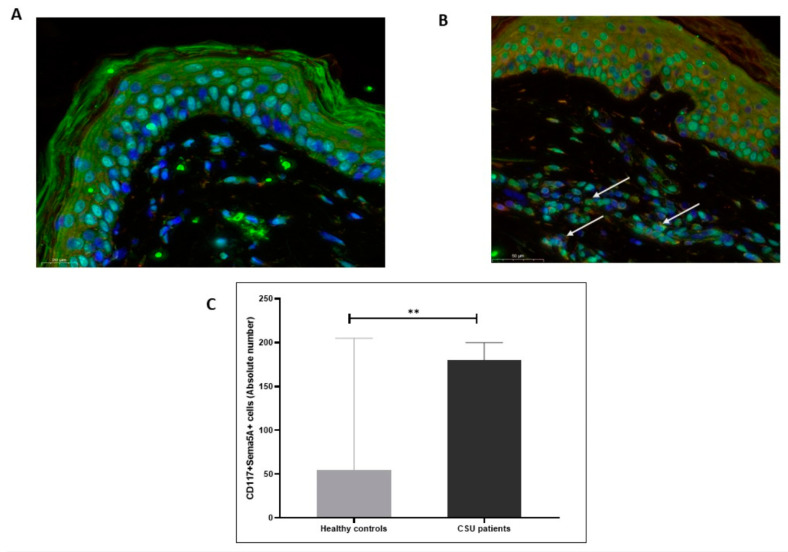
Mast cells in the lesional skin of patients with CSU express Semaphorin5A. Sema5A is expressed by mast cells (CD117+ cells, **B**, white arrows) in the skin of CSU patients compared to healthy controls (**A**). (**C**) shows the results of quantitative analysis of the double-staining of Sema5A/CD117 as evaluated by IMARIS software (as detailed in the “Methods” section). The pictures are 20× magnification. ** statistically significance.

Both CD4+ T cells and mast cells (CD117+) showed the expression of Sema5A in the skin of patients with CSU and healthy control subjects. The skin of patients with CSU had 5-fold higher numbers of Sema5A-expressing CD4+ T cells and 3-fold higher numbers of Sema5A-expressing mast cells, as compared to health control skin (*p* < 0.05; Figure 2 and Figure 3).

**Figure 4:**
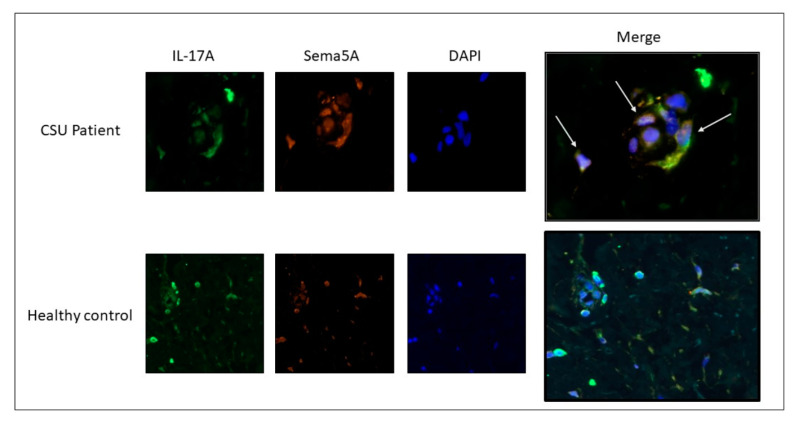
Increased co-expression of Sema5A/IL-17A in CSU skin biopsies. Co-expression of Sema5A and IL-17A was evaluated in skin biopsies taken from healthy subjects and CSU patients. Co-expression of Sema5A/IL-17A is significantly increased in skin biopsies of CSU patients (white arrows). The CSU patient pictures are 40X magnification, and the healthy control pictures are 20× magnification.

Many CD4+ T cells and mast cells in the skin of CSU patients expressed both Sema5A and IL-17A as compared to the skin of healthy individuals, where this co-expression was almost absent (Figure 4).

### 3.2. Sema5A Increases IL-17A Expression in Peripheral CD4+ T Cells

**Figure 5:**
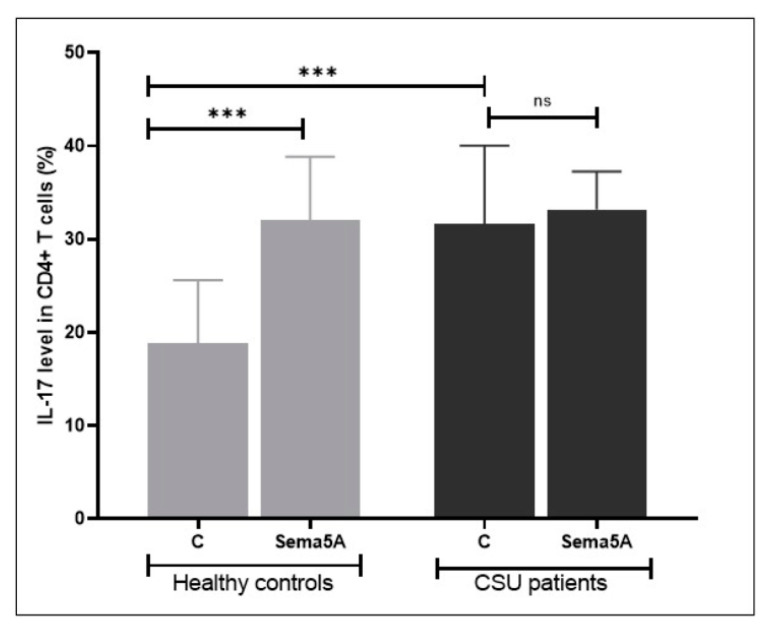
The effect of Sema5A on IL-17 expression in CD4+ T cells. Patients with CSU have higher rates of IL-17-expressing CD4+ T cells as compared to healthy controls. Incubation with Sema5A increases the rate of IL-17-expressing CD4+ T cells in healthy controls, but not in patients with CSU. “ns”—not significance, ***—statistically significance.

Next, we assessed if Sema5A can increase the expression of IL-17A in peripheral activated CD4+ T cells. We found that patients with CSU have significantly higher rates of IL-17A-expressing CD4+ T cells as compared to healthy controls (Figure 5).

Incubation with Sema5A increased the rate of IL-17-expressing CD4+ T cells in healthy controls to that of CSU patients, but did not further increase it in patients with CSU (see also Figure 1).

## 4. Discussion

This is the first study to investigate the role of Sema5A in the pathogenesis of CSU. We show that Sema5A expression is up-regulated in skin T cells and mast cells of patients with CSU and that Sema5A and IL-17A co-localize in the skin of CSU patients. We also demonstrate that Sema5A increases the rates of IL-17A-positive T cells in healthy individuals to those of patients with CSU.

In recent years, numerous studies have investigated the involvement of semaphorins in immune responses. Sema5A, an “immune semaphorin”, comprises transmembrane proteins that exhibit a unique extracellular domain containing seven thrombospondin 1 repeats in addition to the SemaA domain. Sema5A was described to promote angiogenesis by enhancing endothelial cell activation, proliferation, and decreased apoptosis [12]. Receptors that have been reported to bind Sema5A include plexin A1 and plexin B3. Both receptors are expressed on plasmacytoid dendritic cells, NK-cells, and naïve B cells. Knockdown of these receptors led to a significant decrease in Sema5A-induced cytokine secretion. Though they are not expressed on CD4+ T cells, both plexines were reported to be present on CD8+ T cells [13,14]. The stimulation of T cells with Sema5A was previously shown to induce the secretion of Th17/Tc17 cytokines, which strongly suggests that Sema5A can skew T cells towards a Th17 phenotype [9]. The importance of IL-17A in the pathogenesis of CSU was recently demonstrated by our group. When skin biopsies of CSU patients were evaluated, we could clearly show that IL-17A-expressing CD4+ T cells and mast cells are located in close proximity, suggesting this phenomenon to be of mechanistic importance [7]. Thus, the finding of increased expression of Sema5A in the skin of CSU patients in correlation with increased IL-17A expressing CD4+ T cells emphasizes the importance of T cells in the pathogenesis of CSU.

Interestingly, Sema5A increased the rates of IL-17A-positive CD4+ T cells of healthy control subjects to those observed in patients with CSU, but did not further increase them in CSU patients. The latter may be because IL-17A expression in CD4+ T cells of patients with severe CSU is at maximum and can, therefore, not be increased further. This is supported by our previous study, which showed that increased peripheral CD4+ T cells activation and CSU disease severity are correlated. This activation was found to be associated with increased bcl-2 and CD40 ligand expression in activated CD4+ T cells. Of note, increased Sema5A at both the mRNA and protein level is associated with increased bcl-2 expression, which enhances cell migration and invasion into different tissues including the skin [15,16].

Our study has several strengths and limitations. Its major strengths are that we assessed Sema5A and IL-17A colocalization in skin T cells and mast cells of CSU patients and combined this approach with ex vivo analyses of T cell IL-17A expression and how this is affected by Sema5A in CSU patients and healthy controls. Its limitations include the relatively low number of patients analyzed, which did not allow for meaningful comparisons of subpopulations of patients, for example, CSU patients with type I autoimmune CSU vs. type IIb autoimmune CSU.

Taken together, this is the first study on Sema5a in CSU, and our observation of increased co-expression of Sema5A and IL-17A in the skin of CSU patients and not in normal skin supports the notion that IL-17A is a front player in the pathogenesis of CSU. This should encourage further and larger studies on Sema5A in CSU that characterize, in detail, its function, its use as a marker, and its potential as a therapeutic target.
